# Vascular grading of angiogenesis: prognostic significance in breast cancer

**DOI:** 10.1054/bjoc.1999.0924

**Published:** 2000-01-18

**Authors:** S Hansen, D A Grabau, F B Sørensen, M Bak, W Vach, C Rose

**Affiliations:** Departments of Oncology, Pathology and Statistics and Demography, Odense University Hospital, Odense C, DK-5000, Denmark; Department of Pathology, Aarhus County Hospital, Aarhus University Hospital, Aarhus C, DK-8000, Denmark; Oncological Research Centre, Odense University, løvervænget 10-5, Odense C, DK-5000, Denmark

**Keywords:** breast-neoplasms, CD34, neovascularization, observer-variation, prognosis, survival-analysis

## Abstract

The study aimed to evaluate the prognostic value of angiogenesis by vascular grading of primary breast tumours, and to evaluate the prognostic impact of adding the vascular grade to the Nottingham Prognostic Index (NPI). The investigation included 836 patients. The median follow-up time was 11 years and 4 months. The microvessels were immunohistochemically stained by antibodies against CD34. Angiogenesis was graded semiquantitatively by subjective scoring into three groups according to the expected number of microvessels in the most vascular tumour area. The vascular grading between observers was moderately reproduced (κ = 0.59). Vascular grade was significantly associated with axillary node involvement, tumour size, malignancy grade, oestrogen receptor status and histological type. In univariate analyses vascular grade significantly predicted recurrence free survival and overall survival for all patients (*P*< 0.0001), node-negative patients (*P*< 0.0001) and node-positive patients (*P*< 0.0001). Cox multivariate regression analysis showed that vascular grading contributed with independent prognostic value in all patients (*P*< 0.0001). A prognostic index including the vascular grade had clinical impact for 24% of the patients, who had a shift in prognostic group, as compared to NPI, and implied a better prognostic dissemination. We concluded that the angiogenesis determined by vascular grading has independent prognostic value of clinical relevance for patients with breast cancer. © 2000 Cancer Research Campaign


*British Journal of Cancer* (2000) **82**(2), 339–347
© 2000 Cancer Research Campaign
Article no. bjoc.1999.0924

### Vascular grading of angiogenesis: prognostic significance in breast cancer

**S Hansen[1,5], DA Grabau[2,5], FB Sørensen[4], M Bak[2], W Vach[3] and C Rose[1,5]**

Departments of [1]Oncology, [2]Pathology and [3]Statistics and Demography, Odense University Hospital, DK-5000 Odense C, Denmark; [4]Department of Pathology, Aarhus County Hospital, Aarhus University Hospital, DK-8000 Aarhus C, Denmark; [5]Oncological Research Centre, Kløvervænget 10-5, Odense University, DK-5000 Odense C, Denmark

**Summary** The study aimed to evaluate the prognostic value of angiogenesis by vascular grading of primary breast tumours, and to evaluate the prognostic impact of adding the vascular grade to the Nottingham Prognostic Index (NPI). The investigation included 836 patients. The median follow-up time was 11 years and 4 months. The microvessels were immunohistochemically stained by antibodies against CD34. Angiogenesis was graded semiquantitatively by subjective scoring into three groups according to the expected number of microvessels in the most vascular tumour area. The vascular grading between observers was moderately reproduced ($\kappa = 0.59$). Vascular grade was significantly associated with axillary node involvement, tumour size, malignancy grade, oestrogen receptor status and histological type. In univariate analyses vascular grade significantly predicted recurrence free survival and overall survival for all patients ($P < 0.0001$), node-negative patients ($P < 0.0001$) and node-positive patients ($P < 0.0001$). Cox multivariate regression analysis showed that vascular grading contributed with independent prognostic value in all patients ($P < 0.0001$). A prognostic index including the vascular grade had clinical impact for 24% of the patients, who had a shift in prognostic group, as compared to NPI, and implied a better prognostic dissemination. We concluded that the angiogenesis determined by vascular grading has independent prognostic value of clinical relevance for patients with breast cancer. © 2000 Cancer Research Campaign

**Keywords:** breast-neoplasms; CD34; neovascularization; observer-variation; prognosis; survival-analysis

The simple hypothesis about angiogenesis as a prognostic marker is that the intratumoural, leaky vessels may allow the tumour cells to reach the blood stream, and thus increase the metastatic potential and probability of fatal outcome for the patient. This mechanistic explanation may be a simplification of a more complex process, in which the perfusion effect coexists with a paracrine action of the vessels, regulated by growth factors and proteolytic enzymes (Folkman, 1995), modulating the interaction of the stromal component and the tumour cells into a more aggressive metastasizing growth pattern.

Several studies, including nearly 4000 patients, have evaluated intratumoural vessel profiles as a prognostic variable in breast cancer (Bosari et al, 1992; Hall et al, 1992; Horak et al, 1992; Weidner et al, 1992; Khanuja et al, 1993; Toi et al, 1993, 1995; Van Hoef et al, 1993; Fox et al, 1994, 1995*b*; Fregene et al, 1994; Gasparini et al, 1994, 1995; Lipponen et al, 1994; Obermair et al, 1994, 1995; Axelsson et al, 1995; Barbareschi et al, 1995; Bevilacqua et al, 1995; Costello et al, 1995; Goulding et al, 1995; Ogawa et al, 1995; Heimann et al, 1996; Kohlberger et al, 1996; Leek et al, 1996; Morphopoulos et al, 1996; Simpson et al, 1996; Charpin et al, 1997; Karelia et al, 1997; Martin et al, 1997; Acenero et al, 1998). However, these studies have reported contradictory results, without reliable evidence allowing for the clinical use of angiogenesis as a prognostic factor in breast cancer. In addition to the frequently small number of included patients per observational follow-up study, there are differences between studies mainly concerning the applied techniques and the selection of study populations, making comparable analysis of prognostic information difficult. Some authors have discussed the problems of sample size (Harrell et al, 1985; Simon and Altman, 1994), suggesting as a rule of thumb that the number of events should at least be ten times the number of included variables. However, to make the inference reasonable to the general population, the composition of the study population may be just as important as the sample size. A robust inception cohort will be achieved by including all patients in a predefined geographical area and during a defined period.

Ideally a prognosticator should be simple, inexpensive and easy to perform apart from providing independent prognostic value. Vascular grading was introduced by Fox et al (1995*a*) and is a simple method to estimate the degree of tumour angiogenesis. The loss of information by categorization into three groups is compensated by the short period of evaluation per tumour, which is attractive in a clinical setup. Awareness should be given to the reproducibility, because of the subjectivity of this grading system. However, this is a general problem associated with methods assessing angiogenesis by immunohistochemically stained vessel profiles (Hansen et al, 1998).

Knowledge about clinical relevance and application, apart from the statistical significance in a scientific context, is crucial in the evaluation of any prognostic factor. Prognostic indices, calculated from the contribution of relevant variables, provide the individual patient with a prognostic score. A new prognostic variable has clinical impact for patients only when the changes in the prognostic index estimate have consequences for the clinical decision process. However, the clinical decision process may be a hetero-

*Received 28 September 1998*
*Revised 21 April 1999*
*Accepted 13 May 1999*

*Correspondence to:* S Hansen geneous matter. The Nottingham Prognostic Index (NPI) is a validated index (Haybittle et al, 1982; Todd et al, 1987; Galea et al, 1992; Balslev et al, 1994), that has led to clinically useful grouping of breast cancer patients, based solely on tumour size, lymph node state and histological grading of malignancy. Thus, it can be used as a validated predefined tool for clinical decisions in order to investigate the accompanying clinical relevance of a new prognostic factor, i.e. the new prognostic index must spread the survival probabilities of the population to a higher degree.

The aim of the present study was to evaluate the prognostic value of angiogenesis by vascular grading in a large group of breast cancer patients. The reproducibility of the vascular grading technique was examined in a subset of the patients. The prognostic examination was performed by estimating the independent prognostic value of the vascular grading. The accompanying clinical relevance was evaluated by the impact of vascular grading on the prognostic stratification of the patients, compared with what is comprised by the NPI alone.

### MATERIALS AND METHODS

### Sampling of patients

This study was based on the complete population of patients with the diagnosis of breast cancer from a certain geographical area. The region was the primary catchment area of Odense University Hospital, where the patients were operated from 1 January 1980 to 31 December 1990. The inclusion criterion was patients with a primary, unilateral, operable invasive breast carcinoma. The exclusion criteria were patients with distant metastasis at the time of diagnosis, locally advanced disease, inflammatory carcinoma, synchronous bilateral breast cancer and a diagnosis of isolated carcinoma in situ. Women with previous malignant disease, apart from carcinoma in situ of the cervical uterus or skin cancer, were also excluded, as were women who had not had at least one lymph node removed at axillary dissection. Mammographic screening for breast cancer was not performed in the background population during this period. Surgery, adjuvant chemotherapy and radiotherapy were carried out according to the nationwide recommendation of the Danish Breast Cancer Cooperative Group (DBCG) (Andersen and Mouridsen, 1988).

### Number of patients

Of the 1252 women admitted with the suspected diagnosis of breast cancer, the inclusion criterion selected 841 patients. In five patients there was no tumour material available in the archives, leaving 836 patients for the angiogenesis analysis.

A reduced study population ($n$ = 535) was used in the analysis that compared the prognostic impact of NPI with that provided by vascular grading, which was comparable to those patients used in the Nottingham study (Haybittle et al, 1982; Galea et al, 1992). The selection followed the same criteria as in another Danish study that evaluated the NPI (Balslev et al, 1994). Apart from the presence of primary operable breast cancer, the criteria restricted the subset to patients who had had three or more lymph nodes removed from the axilla, who had tumours with histological malignancy grading (i.e. ductal carcinoma, not otherwise specified) and whose age was less than 70 years, such that finally 535 patients was eligible for the comparative analysis. The NPI is defined as: tumour size (in cm) × 0.2 + malignancy grade (1–3) + lymph node status (1–3); lymph node status is 1 if the patients have no metastasis, 2 if one to three metastatic nodes, and 3 if four or more metastatic lymph nodes were detected in the axilla (Galea et al, 1992; Balslev et al, 1994). Grading of histological malignancy followed the grading system of Bloom and Richardson (1957) and WHO guidelines (Scharff and Torloni, 1968).

### Follow-up

Clinical records from departments at which the patients were followed up, and pathology records from Odense University Hospital were reviewed. Patients were followed regularly for 10 years at the Odense University Hospital, according to the DBCG recommendation (Andersen and Mouridsen, 1988), although some older patients were followed by their general practitioner and referred to hospital if recurrence was suspected. Twenty patients moved to other parts of the country. For those patients, the departments providing the follow-up if recurrence was suspected were contacted, and the follow-up information was obtained from clinical records. Two patients moved out of the country and were lost to follow-up. Those patients were censored at the time of the last contact. All the other patients were followed until the closing date of the study, 31 October 1996, or until death, when earlier. The patient recruitment took place over 11 years, and follow-up continued for a further 5 years and 10 months. The maximum possible observed survival times are therefore 16 years and 10 months for the initial patients, and 5 years and 10 months for the last patients. The potential median follow-up time was 11 years and 4 months (136 months).

### End points

The analyses were performed for recurrence-free survival (RFS) and overall survival (OS). The corresponding end points were calculated as the period from surgery to the first recurrence at any site (RFS), or death from any cause (OS). Of the 836 patients, 312 had recurrence, and 381 had died. The evaluation of NPI was performed on OS, as in the study from which the index was derived (Haybittle et al, 1982).

### Immunohistochemistry

All archival blocks from each tumour were initially checked by haematoxylin and eosin-stained sections to select a tumour block with an invasive carcinoma, including the tumour border and as large a cross-sectional area as possible. One 4-μm thick section from each formalin-fixed and paraffin-embedded tumour was mounted on a ChemMate slide (Dako, Denmark). Epitope retrieval for CD34 was performed by microwave heating in 675 ml 10 mM Tris + 0.5 mM EGTA buffer, pH 9, for 25 min with 600 W, cooling in the buffer for 15 min, and rinsing in water for 5 min. The immunostaining procedure was automated, using the ChemMate Peroxidase/DAB kit on the TechMate 1000 instrument (Dako, Denmark). As primary antibody against CD34 we used clone QBEnd/10 (NovoCastra, UK) diluted 1:20 with overnight incubation at 4°C. The primary antibody against the oestrogen receptor (ER) was clone ER1D5 (Dako, Denmark) diluted 1:200 with overnight incubation at 4°C, which was preceded by epitope retrieval by microwave heating with citrate buffer, pH 6. Negative

 © 2000 Cancer Research Campaign controls were produced by omitting the primary antibody, and for each batch a positive control was produced by adding a section with numerous vessel profiles and an oestrogen-positive receptor section respectively.

### Determination of angiogenesis by vascular grading

The amount of immunohistochemically highlighted microvessel profiles were subjectively categorized by vascular grading. The generally accepted criteria for determining a vessel profile (Weidner et al, 1991) were used, including any stained endothelial cell or endothelial-cell cluster that was separate from adjacent microvessels. Vessel lumens were not required for identifying a structure as a microvessel. Microvessels in necrotic or sclerotic areas within a tumour and immediately adjacent areas of unaffected breast tissue were not considered in vessel evaluations. One observer performed the vascular grading by scanning the tumour section at low magnifications, ×4 and ×10 objective lens, thereby finding three separately located tumour areas, where the highest number of discrete microvessels were stained (hot-spots). Each hot-spot area was equivalent to a high power field with a ×25 objective lens and field diameter 0.50 mm. The vascular grading is both influenced by the number of vessel profiles in the initial scanning for hot-spots and by the area of the vessel profiles within the hot-spots in the successive grading process. Thus, given an area with high activity of angiogenesis by many microvessels, the vessel profiles with a larger cross-sectional area or perimeter will contribute more to a high vascular grade. Grade 1 (low angiogenesis) was registered when the combined area of the three hot-spots contained a low amount of endothelial stained microvessel profiles. Grade 1 is typically assigned to tumours without any actual hot-spots. Grade 2 (intermediate angiogenesis) was registered when the combined area of the three hot-spots contained a moderate amount of vessel profiles. Grade 2 is typically assigned to tumours with one very vascular hot-spot and two hot-spots with only a low amount of microvessels. Grade 3 (high angiogenesis) was registered when the combined area of the three hot-spots had numerous vessel profiles with an average large area or perimeter of vessel profiles. Our technique of vascular grading was thus very similar to that of Fox et al (1995*a*), who initially introduced us to the vascular grading. The determination of angiogenesis was performed without knowledge of the prognostic outcome. About one minute was used for vascular grading per tumour (Figure 1).

The reproducibility of the vascular grading was investigated within and between observers. Forty tumours from the initial part of the large material were selected representing the entire measurement scale with 13, 13 and 14 tumours from grades 1, 2 and 3 respectively. The first observer graded the tumours twice at an interval of 1 month, and the second observer graded the same tumours once without knowledge of the first observer's grading.

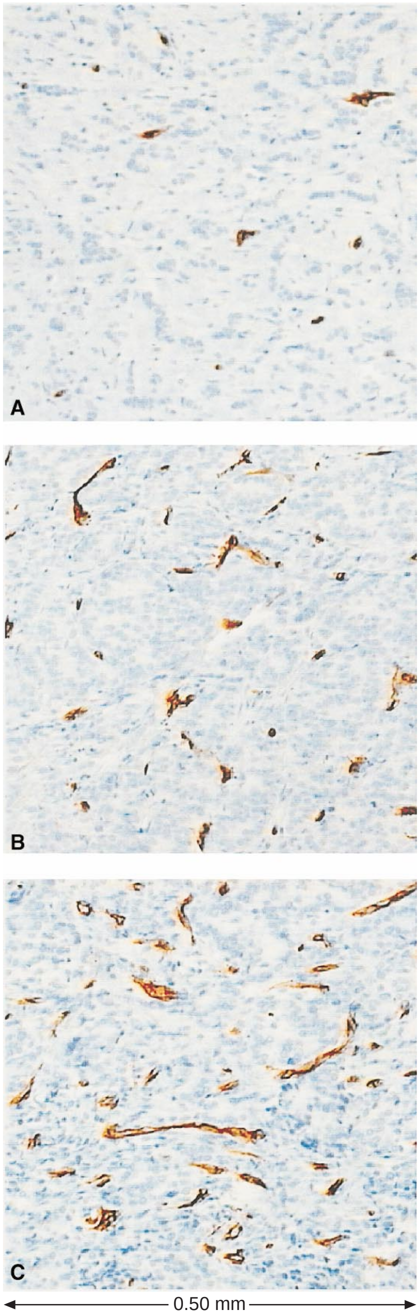

**Figure 1** Examples of three hot-spots with low (**A**), intermediate (**B**) and high (**C**) angiogenesis. The vascular grade of the tumour would be intermediate or grade 2, if the three hot-spots were from the same tumour

### Statistics

The reproducibility of the vascular grading was evaluated by kappa statistics. The association of vascular grading with other variables was tested by the Spearman correlation test for ordinal variables and the $\chi^2$-test for nominal variables. The univariate relationship between the investigated prognostic parameters and follow-up end-points was illustrated by Kaplan–Meier plots for survival probabilities (Kaplan and Meier, 1958). The differences between survival functions were compared by the log-rank test. The multivariate relationship was evaluated by the Cox proportional hazards regression analysis (Cox, 1972). Proportional hazard rates were graphically controlled by log-minus-log-survival plots from the multivariate analysed data stratified by the controlled variable. The ER status did not have proportional hazard rates to fulfil the assumption for the Cox model, hence we stratified the Cox models by ER status. The Cox models were developed by conventional backward selection procedure using a

*© 2000 Cancer Research Campaign*

removal limit of 10% based on the *P*-value from the likelihood ratio statistics. The risk of the categorical covariates was estimated in relation to a reference category, which was always the ‘lowest’ category. Age was an exception, for which the reference was the 40–49 age group, because the category of patients less than 40 years was rather limited in number. The significance of the additional prognostic value of vascular grade to NPI was assessed by a likelihood ratio test. The NPI cut-off points from the initial study were used (Haybittle et al, 1982). The Vascular grade Prognostic Index (VPI) cut-off points were defined to be the level, where the included number of patients in each category was the same as that for the NPI alone. Calculations were performed with SPSS 7.5 (SPSS Inc.)

### RESULTS

### Reproducibility of vascular grading

The distributions of reproducibility data within and between observers are shown in Table 1. Within observer, ten tumours were classified in another grade, but only one had a shift of two grades. Between observers, 11 tumours were classified in another grade, but none had a shift of more than one group. Kappa-statistics with 95% confidence interval (CI) showed within observer κ = 0.62 (0.41–0.83) and between observers κ = 0.59 (0.37–0.81).

**Table 1** Reproducibility data of the vascular grading

| 1st observer 1st scoring | Within observer 1st observer, 2nd scoring | | | Between observer 2nd observer | | | |
|---|---|---|---|---|---|---|---|
| | **1** | **2** | **3** | **1** | **2** | **3** | |
| 1 | 12 | 1 | | 11 | 2 | | **13** |
| 2 | 6 | 5 | 2 | 2 | 7 | 4 | **13** |
| 3 | 1 | | 13 | | 3 | 11 | **14** |
| | 19 | 6 | 15 | 13 | 12 | 15 | |
| | κ = 0.62 | | | κ = 0.59 | | | |
| | 95% CI 0.41–0.83 | | | 95% CI 0.37–0.81 | | | |

### Clinico-pathological data

Table 2 shows the distribution of clinico-pathological characteristics of patients in the study population, and the distribution of vascular grade within the subgroups defined by the different characteristics. Of the 836 patients with immunohistochemically

**Table 2** Description of clinico-pathological data and associated distribution of vascular grade

| Characteristic | All *n* (%) | | Vascular grade 1 | | 2 | | 3 | | Association *P*-value[a] |
|---|---|---|---|---|---|---|---|---|---|
| All patients | 836 | (100) | 392 | (47) | 229 | (27) | 215 | (26) | |
| Age (years) | | | | | | | | | |
| < 40 | 52 | (6) | 29 | (56) | 13 | (25) | 10 | (19) | 0.085 |
| 40–49 | 182 | (22) | 83 | (46) | 58 | (32) | 41 | (22) | |
| 50–59 | 197 | (23) | 98 | (50) | 49 | (25) | 50 | (25) | |
| 60–69 | 199 | (24) | 93 | (47) | 55 | (27) | 51 | (26) | |
| ≥ 70 | 206 | (25) | 89 | (43) | 54 | (26) | 63 | (31) | |
| Menopausal status | | | | | | | | | |
| Premenopausal | 315 | (38) | 158 | (50) | 87 | (28) | 70 | (22) | 0.072 |
| Postmenopausal | 521 | (62) | 234 | (45) | 142 | (27) | 145 | (28) | |
| Lymph node status | | | | | | | | | |
| None | 400 | (48) | 207 | (52) | 110 | (27) | 83 | (21) | <0.001 |
| 1–3 | 277 | (33) | 132 | (48) | 72 | (26) | 73 | (26) | |
| ≥ 4 | 159 | (19) | 53 | (33) | 47 | (30) | 59 | (37) | |
| Tumour size (mm) | | | | | | | | | |
| ≤ 20 | 420 | (50) | 235 | (56) | 108 | (26) | 77 | (18) | <0.001 |
| 21–50 | 361 | (43) | 135 | (38) | 106 | (29) | 120 | (33) | |
| > 50 | 55 | (7) | 22 | (40) | 15 | (27) | 18 | (33) | |
| Malignancy grade | | | | | | | | | |
| I | 140 | (17) | 99 | (71) | 28 | (20) | 13 | (9) | <0.001 |
| II | 316 | (38) | 155 | (49) | 98 | (31) | 63 | (20) | |
| III | 251 | (30) | 61 | (24) | 77 | (31) | 113 | (45) | |
| Other | 129 | (15) | 77 | (60) | 26 | (20) | 26 | (20) | |
| Histological type | | | | | | | | | |
| Ductal | 709 | (85) | 316 | (44) | 204 | (29) | 189 | (27) | 0.004 |
| Lobular | 91 | (11) | 59 | (65) | 18 | (20) | 14 | (15) | |
| Special | 36 | (4) | 17 | (47) | 7 | (20) | 12 | (33) | |
| Oestrogen receptor | | | | | | | | | |
| Negative | 198 | (24) | 45 | (23) | 59 | (30) | 94 | (47) | <0.001 |
| Positive | 638 | (76) | 347 | (54) | 170 | (27) | 121 | (19) | |
| Surgery | | | | | | | | | |
| Mastectomy | 724 | (87) | 328 | (45) | 204 | (28) | 192 | (27) | 0.028 |
| Lumpectomy | 112 | (13) | 64 | (57) | 25 | (22) | 23 | (21) | |
| Adjuvant treatment | | | | | | | | | |
| Chemotherapy | 162 | (19) | 69 | (43) | 47 | (29) | 46 | (28) | 0.222 |
| Endocrine therapy | 139 | (17) | 59 | (42) | 44 | (32) | 36 | (26) | 0.413 |
| Combination | 32 | (4) | 13 | (41) | 8 | (25) | 11 | (34) | 0.319 |
| Radiation therapy | 350 | (42) | 162 | (46) | 95 | (27) | 93 | (27) | 0.680 |

[a]Spearman’s correlation test for the ordinal variables and the $\chi^2$-test for the nominal clinico-pathological variables associated with the vascular grading. Median (range): age 59 years (24–93 years), metastatic lymph nodes 2 (1–47), and of tumour size 20 mm (1–120 mm).

*© 2000 Cancer Research Campaign*

stained tumours, 392 (47%) had vascular grade 1, 229 (27%) grade 2, and 215 (26%) grade 3. High vascular grading was significantly associated with high numbers of axillary lymph node metastases, large tumour size, high malignancy grade and ER-negative tumours. There was also a significant association with histological type, where the lobular carcinoma had many tumours of vascular grade one. The association with the type of surgery did also reach significance, where the smaller tumours after a lumpectomy more often were of vascular grade one. Thus, the median (range) tumour size after mastectomy was 22 mm (1–120 mm), and 15 mm (5–45 mm) after lumpectomy, which was significantly lower ($P$ < 0.0001; Mann–Whitney test). Patients with high age and those being post-menopausal had a tendency to have tumours with higher vascular grades, although these associations were not significant. By contrast, the vascular grading was not related to treatment.

### Univariate analysis

The univariate analysis showed that high vascular grade was a significant indicator of poor prognosis for both RFS and OS (Figure 2), $P < 0.0001$. This relation was documented in both the node-negative ($P < 0.0001$) and node-positive ($P < 0.0001$) patients. The 5- and 10-year survival probability for node-negative, node-positive and all patients is listed in Table 3 for each level of vascular grading.

Apart from vascular grading, the univariate analysis also showed that the prognosis was significantly worsened by a high number of axillary metastatic nodes ($P < 0.0001$ for both RFS and OS), by large tumour size ($P < 0.0001$, both RFS and OS), by high malignancy grade ($P < 0.0001$, both RFS and OS), by high age ($P < 0.0001$, both RFS and OS), for post-menopausal patients ($P < 0.0001$, both RFS and OS), and for ER-negative patients

**Table 3** The recurrence free survival (RFS) and overall survival (OS) probabilities ± s.e.m. in % 5 and 10 years after the diagnosis

| Vascular grade | No. of patients | RFS (%) 5-year | RFS (%) 10-year | OS (%) 5-year | OS (%) 10-year |
|---|---|---|---|---|---|
| All patients ($n$ = 836) | | | | | |
| 1 | 392 | 83 ± 2 | 72 ± 2 | 86 ± 2 | 72 ± 2 |
| 2 | 229 | 63 ± 3 | 51 ± 4 | 71 ± 3 | 52 ± 3 |
| 3 | 215 | 52 ± 4 | 49 ± 4 | 55 ± 3 | 39 ± 4 |
| Node-negative patients ($n$ = 400) | | | | | |
| 1 | 207 | 88 ± 2 | 84 ± 3 | 92 ± 2 | 81 ± 3 |
| 2 | 110 | 70 ± 4 | 61 ± 5 | 81 ± 4 | 60 ± 5 |
| 3 | 83 | 70 ± 5 | 66 ± 6 | 71 ± 5 | 59 ± 6 |
| Node-positive patients ($n$ = 436) | | | | | |
| 1 | 185 | 77 ± 3 | 60 ± 4 | 80 ± 3 | 62 ± 4 |
| 2 | 119 | 57 ± 5 | 41 ± 5 | 62 ± 4 | 44 ± 5 |
| 3 | 132 | 41 ± 4 | 38 ± 5 | 45 ± 4 | 27 ± 4 |

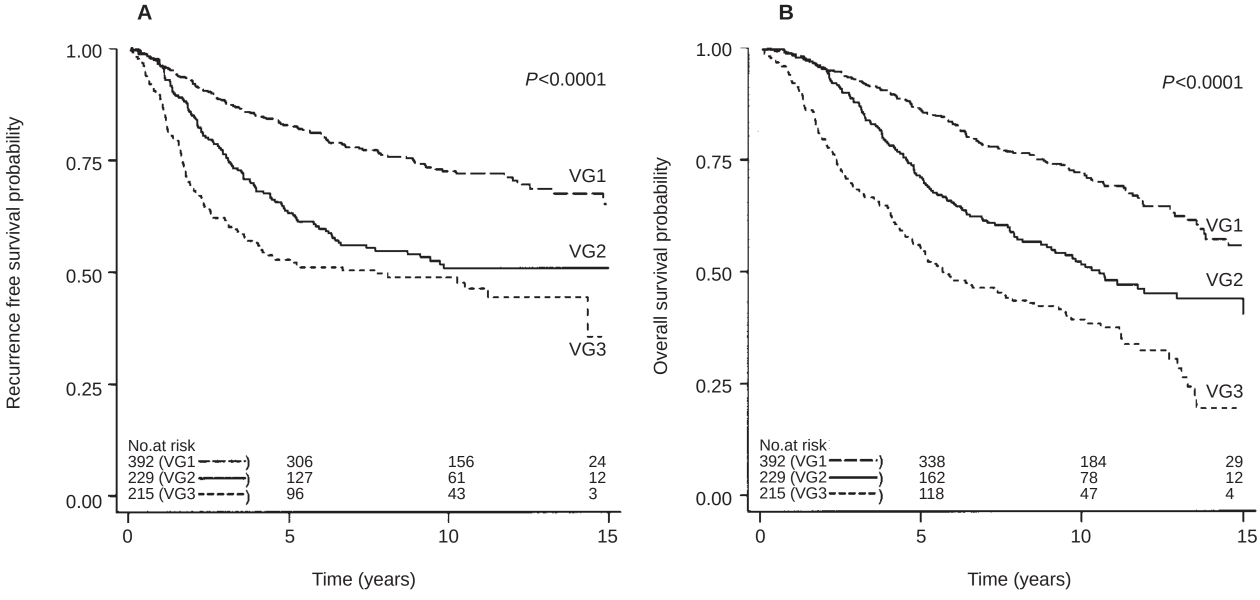

**Figure 2** Kaplan–Meier plots of RFS (A) and OS (B) from the univariate analyses, illustrating the significant differences in survival probabilities between patients with tumours of vascular grades (VG) one, two and three; $n$ = 836

($P$ = 0.0018, RFS; $P$ = 0.0059, OS). The histological type was not a significant prognostic factor.

### Multivariate analysis of vascular grade

The initial Cox model, including all variables mentioned in Table 2, showed that the vascular grade had a statistically significant overall prognostic value ($P$ < 0.0001) independent of all the other variables, and second only to axillary lymph node metastasses in prognostic strength. In this model the risk of dying was 1.71 (95% CI 1.31–2.22) higher with vascular grade 2, and 2.34 (95% CI 1.79–3.05) higher with vascular grade 3, compared with vascular grade 1 and 1.37 (95% CI 1.06–1.77) higher risk with vascular grade 3, compared with vascular grade 2.

Table 4 shows the final model from the Cox multivariate analysis, containing the parameters with significant independent prognostic value. The variables excluded from the initial model during the backward selection procedure were: menopausal status (pre vs post), histological type (ductal vs lobular or special), adjuvant systemic treatment (none vs given), and radiation therapy (none vs given). An independent prognostic value for both RFS and OS, with increased risk, was demonstrated by a high number of axillary lymph node metastases, large tumour size, high malignancy grade and high age. Young age, less than 40 years, was estimated to have a significantly higher risk of recurrence than the 40–49 age group, while the over 70-year-olds did not have a significantly increased risk of recurrence.

The independent prognostic value of the vascular grade was persistent in the subset of node-negative patients, but the risk estimates of vascular grades 2 and 3 were now of the same magnitude. Analysing RFS, the hazard ratios and 95% CI for the vascular grade 2 were 2.27 (95% CI 1.42–3.64), and for grade 3 were 1.89 (95% CI 1.09–3.30). Tumour size was the only other significant factor to predict recurrence in addition to the vascular grade. Analysing OS, the hazard ratios and 95% CI for the vascular grade 2 were 1.79 (95% CI 1.19–2.70), and for grade 3 were 1.82 (95% CI 1.15–2.88). In addition to the vascular grade, the age, and the malignancy grades II and III were the only other significant prognostic factors to predict OS in the node-negative patients. With respect to the node-positive subgroup the same calculations showed that for RFS, the hazard ratios and 95% CI for the vascular grade 2 were 1.80 (95% CI 1.25–2.58), and for grade 3 were 2.64 (95% CI 1.84–3.79). Analysing OS, the hazard ratios and 95% CI for the vascular grade 2 were 1.63 (95% CI 1.16–2.29), and for grade 3 were 2.58 (95% CI 1.85–3.61). The malignancy grade had no significant prognostic value, while number of metastatic lymph nodes, tumour size and age, were of significant prognostic impact for both RFS and OS.

### Angiogenesis in relation to the NPI

The NPI is able to split patients with breast cancer into three groups with significantly different prognosis (Haybittle et al, 1982; Galea et al, 1992). Figure 3 shows the OS probabilities in the three NPI-groups, and of the three vascular grades in the reduced study group ($n$ = 535). The independent prognostic value given by the Cox analysis for the OS could also be shown in the reduced study population. Repeating all analyses, the same variables were significant, and the risk estimates were of the same magnitude (results not shown).

Table 5 shows a Cox model with NPI alone (model A) and combined with the vascular grade (model B), both providing significant prediction of prognosis. The data was fitted significantly better by model B than in model A ($P$ < 0.0001). The vascular grade thus added independent prognostic value to that provided by the NPI alone, and a combined index 0.54×NPI + 0.45×vascular grade could be built. As both effects are of similar magnitude, we suggest to use simply the new index:

Vascular Grade Prognostic Index (VPI) = NPI + Vascular Grade

The VPI value was calculated for the individual patient. The patients were then divided into three groups, each with a number of patients of comparable size to that of the three corresponding isolated NPI groups. In Table 6, the two index groups are cross-

**Table 4** The Cox multivariate analysis estimated the independent prognostic values by hazard ratios (HR) and 95% CI for both recurrence free survival (RFS) and overall survival (OS)

| | | RFS | | OS | |
|---|---|---|---|---|---|
| **Variables** | **Categories** | ***P*-value** | **HR (95% CI)** | ***P*-value** | **HR (95% CI)** |
| Age (years) | 40–49 | 0.0158 | 1.00 | <0.0001 | 1.00 |
| | < 40 | | 1.73 (1.04–2.88) | | 1.11 (0.61–2.04) |
| | 50–59 | | 1.68 (1.19–2.38) | | 1.92 (1.34–2.77) |
| | 60–69 | | 1.74 (1.23–2.46) | | 2.31 (1.61–3.31) |
| | ≥ 70 | | 1.41 (0.99–2.02) | | 4.27 (3.05–5.98) |
| Lymph node status | None | <0.0001 | 1.00 | <0.0001 | 1.00 |
| | 1–3 | | 1.40 (1.06–1.85) | | 1.37 (1.07–1.76) |
| | ≥ 4 | | 3.57 (2.66–4.81) | | 3.55 (2.71–4.63) |
| Tumour size | per cm | 0.0009 | 1.12 (1.05–1.19) | 0.0062 | 1.09 (1.02–1.16) |
| Malignancy grade | Grade I, ductal | 0.1915 | 1.00 | 0.0493 | 1.00 |
| | Grade II, ductal | | 1.52 (1.00–2.31) | | 1.57 (1.08–2.28) |
| | Grade III, ductal | | 1.60 (1.01–2.53) | | 1.80 (1.19–2.74) |
| | Non-ductal | | 1.61 (0.99–2.60) | | 1.50 (0.98–2.31) |
| Vascular grade | Grade 1 | <0.0001 | 1.00 | <0.0001 | 1.00 |
| | Grade 2 | | 1.92 (1.45–2.55) | | 1.70 (1.31–2.20) |
| | Grade 3 | | 2.40 (1.78–3.24) | | 2.32 (1.77–3.03) |

The variables are presented as indicators with risk estimates relative to the basic category, where the risk is set to one, e.g. basic category for age is 40–49 years. $P$-values for the single variables are shown at the baseline category. All 836 patients are included.

 © 2000 Cancer Research Campaign

**Table 5** Cox multivariate analysis including Nottingham Prognostic Index (NPI) and vascular grade

| Variables | Model A β | Model A *P* | Model B β | Model B *P* |
|---|---|---|---|---|
| NPI (continuous) | 0.61 | <0.0001 | 0.54 | <0.0001 |
| Vascular grade (1 vs 2 vs 3) | – | – | 0.45 | <0.0001 |
| –2 log likelihood | 2292.6 | | 2266.2 | |
| Difference (A vs B) | | | 26.4 | |
| *P*-value; $\chi^2$-test (A vs B) | | | < 0.0001 | |

Highly significant regression coefficients (β) were obtained for both variables, and model B had a significantly better prediction of prognosis than that provided by model A alone. (535 patients analysed).

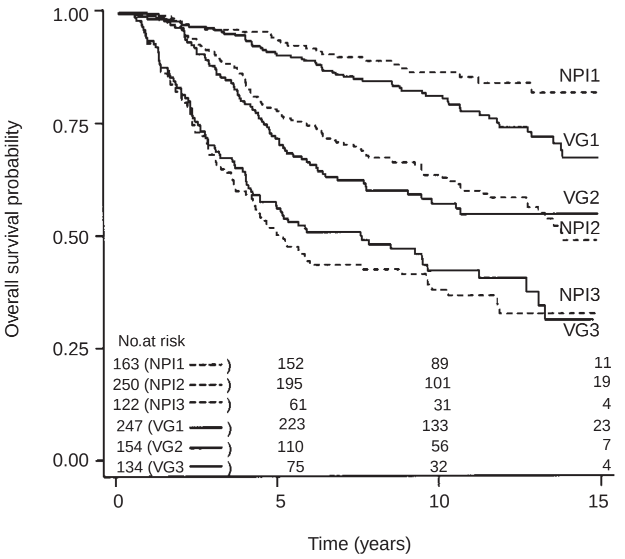

**Figure 3** Kaplan–Meier plots of overall survival probabilities in the three vascular grades (VG), and in the three Nottingham Prognostic Index groups (NPI). $n$ = 535 for both variables. This study population included patients less than 70 years, who had had three or more lymph nodes removed from the axilla, and who had a malignancy grading assigned to their tumours (i.e. ductal carcinoma, not otherwise specified)

**Table 6** Cross-tabulation of NPI-groups and VPI-groups, showing the exchange of patients by adding the independent prognostic value from the vascular grade to that of the NPI

| | | VPI 1 | VPI 2 | VPI 3 | |
|---|---|---|---|---|---|
| NPI | 1 | 132 | 31 | | 163 |
| | 2 | 35 | 183 | 32 | 250 |
| | 3 | | 32 | 90 | 122 |
| | | 167 | 246 | 122 | 535 |

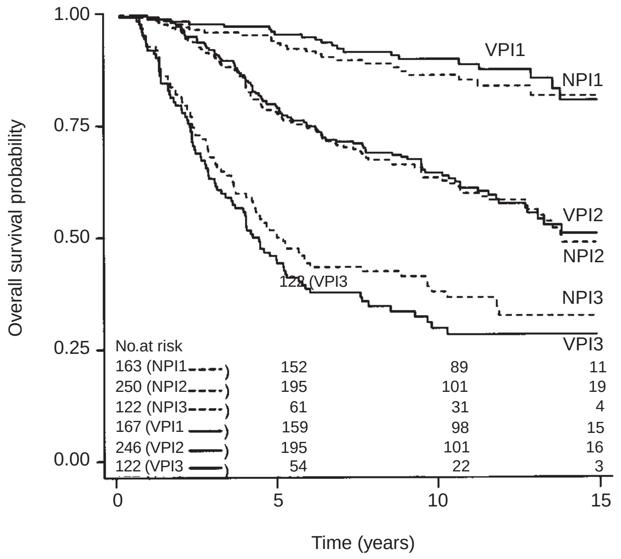

**Figure 4** Kaplan–Meier plot comparing the overall survival probabilities from patients in the three groups of the Vascular grade Prognostic Index (VPI) with the three groups of the Nottingham Prognostic Index (NPI). It was intended to have the same number of patients in group one for both variables, although not the same patients. The same constraints apply for groups two and three. $n$ = 535 for both variables tabulated, showing that 31 patients from NPI group one were removed to VPI group two, and that the 35 new patients included in VPI group one were taken from NPI group two. If group one is used to decide not to offer adjuvant treatment and group two to do so, as suggested by the Nottingham group (Galea et al, 1992), then the treatment decision would be changed by incorporating vascular grade in the above example for 66 patients. In particular, 31 patients previously treated would now receive no treatment, and 35 patients previously not treated would receive treatment. The exchange between the second and third groups was of the same magnitude. A total of 24% of the patients (31+35+32+32) changed prognostic group. Figure 4 illustrates the gain in prognostic discrimination going from NPI to VPI. The patients in group one selected by the VPI showed a somewhat better survival than the comparable group selected by the NPI. Furthermore, VPI group three had a poorer prognosis than NPI group three.

### DISCUSSION

Our study focuses on three objectives. First, the reliability of vascular grading to assess angiogenesis in breast carcinomas. Second, the prognostic value of the vascular grade in breast carcinomas. Third, the practical clinical relevance of introducing the prognostic value of the vascular grade, compared with therapeutic decisions based on the predefined prognostic algorithm of the NPI.

### Reproducibility of vascular grading

Assessing angiogenesis by vascular grading is attractive because it takes about 1 min per slide. However, the drawbacks are loss of information due to categorization, and only a moderate reproducibility due to subjectivity, just like other hot-spot techniques for assessing angiogenesis (Hansen et al, 1998). The grading approach with three groups has been used earlier (Fox et al, 1995*a*), although the definitions of classifications may be slightly

© 2000 Cancer Research Campaign different. The present evaluation documented a substantial intraobserver reproducibility, $\kappa$ = 0.62, and a moderate interobserver reproducibility, $\kappa$ = 0.59, representing an acceptable reproducibility (Landis and Koch, 1977; Svanholm et al, 1989). Other workers have previously reported substantial intraobserver reproducibility, $\kappa$ = 0.66, by using only two grades (Fox et al, 1997). Using the same design of categorizing the present data, by comparing grade 1 plus 2 with grade 3 we obtained an optimized intraobserver reproducibility, $\kappa$ = 0.84.

Another method for estimating angiogenesis is by the Chalkley count technique. The vascular grade has previously been related to the Chalkley count, $P$ = 0.001 (Fox et al, 1995*a*). The Chalkley count by using a 25-point ocular grid has a more objective classification criteria for the categorizing of for example three groups, and therefore may be a more reliable method to introduce into routine practice. On the other hand, the Chalkley count has also been shown to have only a moderate reproducibility due to the initial subjective selection of the hot-spot areas (Hansen et al, 1998). Furthermore, the prognostic value of the vascular grade is not necessarily related to the prognostic value of the Chalkley count, and it is therefore of interest to estimate the prognostic value of the vascular grade, which has not been published before.

The archival material was stored in paraffin after varying fixation times, which were unlikely to influence the estimates. In the pilot studies evaluating immunohistochemical staining optimization, we did not find that the staining reaction for CD34 depended on fixation times. The storage period of the paraffin blocks is not likely to influence the immunostaining of vessel profiles. After initial staining in a pilot sample with FVIII-ra, CD31, and CD34, we decided to use CD34, because it stained more microvessel profiles than FVIII-ra. In practice, antibodies to CD31 could probably be used as well, though they occasionally stain inflammatory cells. Using an automated immunostainer further reduced the variation due to laboratory techniques.

### Prognostic value

The study showed that the vascular grade had an independent prognostic value regarding both RFS and OS. We believe that we can infer the results to the general population, because we had a predefined sampling of the study population with few missing data, a long follow-up, and a sufficient number of events. The differences in survival probabilities for the patients with different vascular grades were not likely to be due to treatment, since the treatment was given within each group of vascular grade to the same proportion of patients as in the entire study population, indicating no association with the vascular grade from any of the treatment modalities.

The role of angiogenesis as a prognosticator has been evaluated in many series of patients with breast cancer (Fox, 1997); it has been reported to have both prognostic value and the contrary. The difficulties in extracting meaningful information from the literature regarding the prognosis are not confined to conflicting results; they come mostly from the different techniques applied and the uncertainty about the selection of the study populations, as demonstrated by the heterogeneous composition of published studies. In our study we accentuate the high quality of the study population sample. This was achieved by a population-based identification of the patients.

### Angiogenesis in relation to the NPI

The NPI has been recommended for selecting patients for individualized adjuvant treatments according to the assigned prognostic group (Galea et al, 1992). The reason for using the NPI in our study was to have predefined validated prognostic criteria for making therapeutic decisions. These plain criteria are chosen, because decisions about advice and adjuvant treatment may differ from department to department due to other factors such as predictive factors, expectation about local control, or even personal preferences. The vascular grade is not supposed to replace the NPI, since the prognostic information is independent of and additive to the basic prognostic variables included in the NPI. Inclusion in the NPI of the prognostic value derived from the vascular grade would change the prognostic risk estimate for many patients in our study; for 24% of them to the extent that the NPI-based decision about advice and adjuvant treatment would be changed. The survival probability curves have a broader spread after the re-stratification based on incorporation of the vascular grade into a combined prognostic index. This better distinction of the prognosis demonstrates the clinical relevance of the vascular grade.

In conclusion, vascular grading is a fast and easy-to-perform estimate of angiogenesis, likely to be acceptable to pathologists in general, but the reliability of the estimate is no better than other methods assessing angiogenesis from hot-spot. Loss of information due to the classification of the variable may be possible. This moderate reliability may be accepted, if the prognostic value is strong. Angiogenesis determined by vascular grade has independent prognostic value of clinical relevance for patients with breast cancer. The strong prognostic value of the vascular grade advocates for the possibility of clinical implementation, although a confirmatory study must be carried out. The vascular grading is influenced not only by the number of vessel profiles, but also the area of the vessel profiles. Future research on angiogenesis methods should look upon more objective classification criteria including the surface density and the volume density of the vessels, and the importance of the mean area of the single vessel profile. Objective classification criteria would facilitate the acceptability of the scoring system, but the cost and time consumption of complicated methodological readings would not be beneficial for clinical diagnostic use.

### ACKNOWLEDGEMENTS

We thank Stephen B Fox for initial training in assessment of angiogenesis by the hot-spot approach. The technical assistance of Ole Nielsen and Maj-Britt Berg is greatly appreciated. This research was supported by grants from The Danish Cancer Society, King Chr X Foundation, Novo Nordic Foundation, and Odense University.

### REFERENCES

Acenero MJF, Gallego MG, Ballesteros PA and Gonzales JF (1998) Vascular density as a prognostic indicator for invasive ductal breast carcinoma. *Virchows Arch* **432:** 113–117

Andersen KW and Mouridsen HT (1988) Danish Breast Cancer Cooperative Group (DBCG). A description of the register of the nation-wide programme for primary breast cancer. *Acta Oncol* **27**: 627–647

Axelsson K, Ljung B-ME, Moore II DH, Thor AD, Chew KL, Edgerton SM, Smith HS and Mayall BH (1995) Tumor angiogenesis as a prognostic assay for invasive ductal breast carcinoma. *J Natl Cancer Inst* **87**: 997–1008

 © 2000 Cancer Research Campaign

Balslev I, Axelsson CK, Zedeler K, Rasmussen BB, Carstensen B and Mouridsen HT (1994) The Nottingham Prognostic Index applied to 9,149 patients from the studies of the Danish Breast Cancer Cooperative Group (DBCG). *Breast Cancer Res Treat* **32**: 281–290

Barbareschi M, Weidner N, Gasparini G, Morelli L, Forti S, Eccher C, Fina P, Caffo O, Leonardi E, Mauri F, Bevilacqua P and Palma PD (1995) Microvessel density quantification in breast carcinomas. Assessment by light microscopy vs. a computer-aided image analysis system. *Appl Immunohistochem* **3**: 75–84

Bevilacqua P, Barbareschi M, Verderio P, Boracchi P, Caffo O, Palma PD, Meli S, Weidner N and Gasparini G (1995) Prognostic value of intratumoral microvessel density, a measure of tumor angiogenesis, in node negative breast carcinoma – results of a multiparametric study. *Breast Cancer Res Treat* **36**: 205–217

Bloom HJG and Richardson WW (1957) Histological grading and prognosis in breast cancer. A study of 1409 cases of which 359 have been followed for 15 years. *Br J Cancer* **11**: 359–377

Bosari S, Lee AK, DeLellis RA, Wiley BD, Heatley GJ and Silverman ML (1992) Microvessel quantitation and prognosis in invasive breast carcinoma. *Hum Pathol* **23**: 755–761

Charpin C, Garcia S, Bouvier C, Martini F, Andrac L, Bonnier P, Lavaut MN and Allasia C (1997) CD31/PECAM automated and quantitative immunocytochemical assays in breast carcinomas: correlation with patient follow-up. *Am J Clin Pathol* **107**: 534–541

Costello P, McCann A, Carney DN and Dervan PA (1995) Prognostic significance of microvessel density in lymph node negative breast carcinoma. *Hum Pathol* **26**: 1181–1184

Cox DR (1972) Regression models and life tables. *J Roy Stat Soc* **34**: 187–220

Folkman J (1995) Tumor angiogenesis. In: *The Molecular Basis of Cancer*, Mendelsohn J, Howley PM, Israel MA and Liotta LA (eds), pp. 206–232. WB Saunders: Philadelphia

Fox SB (1997) Tumour angiogenesis and prognosis. *Histopathology* **30**: 294–301

Fox SB, Leek RD, Smith K, Hollyer J, Greenall M and Harris AL (1994) Tumor angiogenesis in node-negative breast carcinomas – relationship with epidermal growth factor receptor, estrogen receptor, and survival. *Breast Cancer Res Treat* **29**: 109–116

Fox SB, Leek RD, Weekes MP, Whitehouse RM, Gatter KC and Harris AL (1995*a*) Quantitation and prognostic value of breast cancer angiogenesis: comparison of microvessel density, Chalkley count, and computer image analysis. *J Pathol* **177**: 275–283

Fox SB, Turner GDH, Leek RD, Whitehouse RM, Gatter KC and Harris AL (1995*b*) The prognostic value of quantitative angiogenesis in breast cancer and role of adhesion molecule expression in tumour endothelium. *Breast Cancer Res Treat* **36**: 219–226

Fox SB, Leek RD, Bliss J, Mansi JL, Gusterson B, Gatter KC and Harris AL (1997) Association of tumor angiogenesis with bone marrow micrometastases in breast cancer patients. *J Natl Cancer Inst* **89**: 1044–1049

Fregene TA, Khanuja PS, Gimotty PA, Kellogg C, George J and Pienta KJ (1994) The relationship of microvessel counts to tumor size, estrogen receptor status, lymph node metastasis, and disease-free survival in patients with stage I and II breast cancer. *Int J Oncol* **5**: 1437–1445

Galea MH, Blamey RW, Elston CE and Ellis IO (1992) The Nottingham Prognostic Index in primary breast cancer. *Breast Cancer Res Treat* **22**: 207–219

Gasparini G, Weidner N, Bevilacqua P, Maluta S, Dalla Palma P, Caffo O, Barbareschi M, Boracchi P, Marubini E and Pozza F (1994) Tumor microvessel density, p53 expression, tumor size, and peritumoral lymphatic vessel invasion are relevant prognostic markers in node-negative breast carcinoma. *J Clin Oncol* **12**: 454–466

Gasparini G, Barbareschi M, Boracchi P, Bevilacqua P, Verderio P and Dalla Palma PM-S (1995) 67-kDa laminin-receptor expression adds prognostic information to intra-tumoral microvessel density in node-negative breast cancer. *Int J Cancer* **60**: 604–610

Goulding H, Abdul Rashid NFN, Robertson JF, Bell JA, Elston CW, Blamey RW and Ellis IO (1995) Assessment of angiogenesis in breast carcinoma: an important factor in prognosis? *Hum Pathol* **26**: 1196–1200

Hall NR, Fish DE, Hunt N, Goldin RD, Guillou PJ and Monson JR (1992) Is the relationship between angiogenesis and metastasis in breast cancer real? *Surg Oncol* **1**: 223–229

Hansen S, Grabau DA, Rose C, Bak M and Sørensen FB (1998) Angiogenesis in breast cancer. A comparative study of the observer variability of methods for determining microvessel density. *Lab Invest* **78**: 1563–1573

Harrell FE Jr, Lee KL, Matchar DB and Reichert TA (1985) Regression models for prognostic prediction: advantages, problems, and suggested solutions. *Cancer Treat Rep* **69**: 1071–1077

Haybittle JL, Blamey RW, Elston CW, Johnson J, Doyle PJ, Campbell FC, Nicholson RI and Griffiths K (1982) A prognostic index in primary breast cancer. *Br J Cancer* **45**: 361–366

Heimann R, Ferguson D, Powers C, Recant WM, Weichselbaum RR and Hellman S (1996) Angiogenesis as a predictor of long-term survival for patients with node-negative breast cancer. *J Natl Cancer Inst* **88**: 1764–1769

Horak ER, Leek R, Klenk N, LeJeune S, Smith K, Stuart N, Greenall M, Stepniewska K and Harris AL (1992) Angiogenesis, assessed by platelet/endothelial cell adhesion molecule antibodies, as indicator of node metastases and survival in breast cancer. *Lancet* **340**: 1120–1124

Kaplan EL and Meier P (1958) Nonparametric estimation from incomplete observations. *J Am Stat Assoc* **53**: 457–481

Karelia NH, Patel DD, Balar DB, Desai NS, Patel SU, Dave K and Shah GB (1997) Prognostic significance of tumor angiogenesis in advanced breast carcinoma: an Indian experience. *Neoplasma* **44**: 163–166

Khanuja PS, Gimotty P, Fregene T, George J and Pienta KJ (1993) Angiogenesis quantitation as a prognostic factor for primary breast carcinoma 2 cms or less. In: *Adjuvant Therapy of Cancer*, Salmon SE (ed), pp. 226–232. Lippincott: Philadelphia

Kohlberger PD, Obermair A, Sliutz G, Heinzl H, Koelbl H, Breitenecker G, Gitsch G and Kainz C (1996) Quantitative immunohistochemistry of factor VIII-related antigen in breast carcinoma. *Am J Clin Pathol* **105**: 705–710

Landis JR and Koch GG (1977) The measurement of observer agreement for categorical data. *Biometrics* **33**: 159–174

Leek RD, Lewis CE, Whitehouse R, Greenall M, Clarke J and Harris AL (1996) Association of macrophage infiltration with angiogenesis and prognosis in invasive breast carcinoma. *Cancer Res* **56**: 4625–4629

Lipponen P, Ji H, Aaltomaa S and Syrjanen K (1994) Tumour vascularity and basement membrane structure in breast cancer as related to tumour histology and prognosis. *J Cancer Res Clin Oncol* **120**: 645–650

Martin L, Green B, Renshaw C, Lowe D, Rudland P, Leinster SJ and Winstanley J (1997) Examining the technique of angiogenesis assessment in invasive breast cancer. *Br J Cancer* **76**: 1046–1054

Morphopoulos G, Pearson M, Ryder WD, Howell A and Harris M (1996) Tumour angiogenesis as a prognostic marker in infiltrating lobular carcinoma of the breast. *J Pathol* **180**: 44–49

Obermair A, Czerwenka K, Kurz C, Kaider A and Sevelda P (1994) Tumoral vascular density in breast tumors and their effect on recurrence-free survival. *Chirurgie* **65**: 611–615

Obermair A, Kurz C, Czerwenka K, Thoma M, Kaider A, Wagner T, Gitsch G and Sevelda P (1995) Microvessel density and vessel invasion in lymph-node-negative breast cancer: effect on recurrence-free survival. *Int J Cancer* **62**: 126–131

Ogawa Y, Chung Y-S, Nakata B, Takatsuka S, Maeda K, Sawada T, Kato Y, Yoshikawa K, Sakurai M and Sowa M (1995) Microvessel quantitation in invasive breast cancer by staining for factor VIII-related antigen. *Br J Cancer* **71**: 1297–1301

Scharff RW and Torloni H (1968) Histological typing of breast tumours. International histological classification of tumours, No. 2. WHO; Geneva

Simon R and Altman DG (1994) Statistical aspects of prognostic factor studies in oncology. *Br J Cancer* **69**: 979–985

Simpson JF, Ahn C, Battifora H and Esteban JM (1996) Endothelial area as a prognostic indicator for invasive breast carcinoma. *Cancer* **77**: 2077–2085

Svanholm H, Starklint H, Gundersen HJ, Fabricius J, Barlebo H and Olsen S (1989) Reproducibility of histomorphologic diagnoses with special reference to the kappa statistic. *APMIS* **97**: 689–698

Todd JH, Dowle C, Williams MR, Elston CW, Ellis IO, Hinton CP, Blamey RW and Haybittle JL (1987) Confirmation of a prognostic index in primary breast cancer. *Br J Cancer* **56**: 489–492

Toi M, Kashitani J and Tominaga T (1993) Tumor angiogenesis is an independent prognostic indicator in primary breast carcinoma. *Int J Cancer* **55**: 371–374

Toi M, Inada K, Suzuki H and Tominaga T (1995) Tumor angiogenesis in breast cancer: its importance as a prognostic indicator and the association with vascular endothelial growth factor expression. *Breast Cancer Res Treat* **36**: 193–204

Van Hoef ME, Knox WF, Dhesi SS, Howell A and Schor AM (1993) Assessment of tumour vascularity as a prognostic factor in lymph node negative invasive breast cancer. *Eur J Cancer* **29A**: 1141–1145

Weidner N, Folkman J, Pozza F, Bevilacqua P, Allred EN, Moore DH, Meli S and Gasparini G (1992) Tumor angiogenesis: a new significant and independent prognostic indicator in early-stage breast carcinoma. *J Natl Cancer Inst* **84**: 1875–1887

*© 2000 Cancer Research Campaign*
